# Essential oils as capsule disruptors: enhancing antibiotic efficacy against multidrug-resistant *Klebsiella pneumoniae*

**DOI:** 10.3389/fmicb.2024.1467460

**Published:** 2024-08-30

**Authors:** Azza SalahEldin El-Demerdash, Rihaf Alfaraj, Faten A. Farid, Mohamed H. Yassin, Abdulrahman M. Saleh, Ghada E. Dawwam

**Affiliations:** ^1^Laboratory of Biotechnology, Department of Microbiology, Agricultural Research Center (ARC), Animal Health Research Institute (AHRI), Zagazig, Egypt; ^2^Department of Pharmaceutics, College of Pharmacy, King Saud University, Riyadh, Saudi Arabia; ^3^Botany and Microbiology Department, Faculty of Science, Benha University, Benha, Egypt; ^4^Pharmaceutical Medicinal Chemistry and Drug Design Department, Faculty of Pharmacy (Boys), Al-Azhar University, Cairo, Egypt; ^5^Epidemiological Surveillance Unit, Aweash El-Hagar Family Medicine Center, MOHP, Mansoura, Egypt

**Keywords:** antibacterial activity, bioactive compounds, capsule gene expression, *Klebsiella pneumoniae*, tea tree oil, thyme oil

## Abstract

**Background:**

Multidrug-resistant *Klebsiella pneumoniae* (MDR-*KP*) poses a significant global health threat due to its involvement in severe infections and high mortality rates. The emergence of MDR strains necessitates the exploration of alternative therapeutic strategies.

**Methods:**

*K. pneumoniae* isolates were obtained from human and animal sources. Antibacterial susceptibility testing was performed, followed by the evaluation of essential oil activity through inhibition zone, MIC, and MBC determinations. Checkerboard assays were conducted to assess synergistic effects with amikacin. Gene expression analysis and transmission electron microscopy were employed to elucidate the mechanisms of action. Molecular docking studies were performed to identify potential binding targets of bioactive compounds.

**Results:**

*Klebsiella pneumoniae* was isolated from 25 of the100 samples examined, representing a prevalence rate of 25%. All isolates were found to be multidrug-resistant. Tea tree and thyme essential oils exhibited potent antibacterial activity and synergistic effects with amikacin. Notably, these combinations significantly downregulated the expression of key capsule virulence genes (*wcaG*, *rmpA*, *magA*, *uge*, and *wabG*), suggesting a novel mechanism for enhancing amikacin efficacy. Transmission electron microscopy revealed disrupted cell integrity in MDR-KP cells treated with the combinations. Molecular docking analysis identified Terpinen-4-ol, Farnesol, 1,4-Dihydroxy-p-menth-2-ene, and 7-Oxabicyclo [4.1.0] heptane as potential bioactive compounds responsible for the observed effects.

**Conclusion:**

By effectively combating MDR-KP, this research holds promise for reducing antibiotic resistance, improving treatment outcomes, and ultimately enhancing potential care.

## Introduction

An opportunistic bacterium known as *Klebsiella pneumoniae* infects hospitalized or immunocompromised patients (Gorrie et al., 2017). Similarly, in animals, such as bovine, *K. pneumoniae* is a major causative agent of mastitis, leading to economic losses and public health concerns (Opoku-Temeng et al., 2019; Yang et al., 2019; El-Demerdash et al., 2023b). This rod-shaped, encapsulated, non-motile member of the Enterobacteriaceae family is gram-negative (Nasr et al., 2016).

In medical settings, multidrug-resistant *Klebsiella pneumoniae* (MDR-*KP*) is a significant contributor to the high rates of morbidity and death in patients with severe infections (Lee et al., 2016). Infections caused by MDR-*KP* strains are increasingly common worldwide (Abdelaziz et al., 2013). This issue may have originated in underdeveloped nations due to the overuse of antibiotics, incorrect prescriptions, unnecessary testing, and medication abuse (Nordmann et al., 2009; Shalaby et al., 2021; Megahed et al., 2023).

The global emergence of MDR-*KP*, often causing hospital-acquired infections, necessitates new therapeutic strategies (Davies and Davies, 2010; Essawi et al., 2020; Abd El-Emam et al., 2023; Hashem et al., 2024). The spread of strains producing extended-spectrum beta-lactamases (ESBLs) and carbapenems underscores the urgent need for alternative treatments (Hawkey et al., 2018; Ebrahem et al., 2023). Essential oils (EOs), volatile compounds extracted from plants, have emerged as promising antimicrobial agents due to their diverse mechanisms of action, including disruption of cell membranes, inhibition of protein synthesis, and interference with DNA replication (Birhan et al., 2011; Rybicki et al., 2012; Dhama et al., 2015).

Numerous investigations have demonstrated that specific phytochemicals, like those found in EOs, and antibiotics, when used together, produce synergistic effects. These combinations can inhibit folate biosynthesis, DNA/protein synthesis, and disrupt cell permeability and cell wall (Dhami, 2013). Additionally, EOs can further weaken bacteria by preventing them from synthesizing essential macromolecules like DNA, RNA, proteins, and polysaccharides (Konaté et al., 2012). This multi-pronged attack offered by the combined approach proves to be highly beneficial in combating multidrug-resistant (MDR) bacteria. By targeting multiple bacterial processes simultaneously, EOs and antibiotics can effectively overcome resistance mechanisms employed by MDR pathogens, leading to more efficient treatment (Abd El-Kalek and Mohamed, 2012).

Previous research has explored the antibacterial properties of EOs against *Klebsiella pneumoniae* (Mohamed et al., 2018; Diniz et al., 2023). However, no previous studies have examined the ability of essential oils to specifically target the capsule of *K. pneumoniae*, a crucial virulence factor associated with immune evasion.

The capsule that surrounds the surface of *K. pneumoniae* serves as the primary virulence factor associated with its viscous phenotype. It typically provides a defensive rather than offensive resistance to bactericidal activities (Zhu et al., 2021). *K. pneumoniae* utilizes capsules to prevent bacteria from binding, thus evading phagocytosis, complement, antimicrobial peptides, and specific antibodies. However, instances of active suppression and attack on immune cells through capsules are rarely reported (Paczosa and Mecsas, 2016). The chromosomal capsular polysaccharide synthesis (cps) region encodes the genes responsible for capsule production. The cps cluster comprises 27 genes, including *rmpA, wcaG, magA, uge*, and *wabG* (Ernst et al., 2020).

Therefore, this study addresses this gap by investigating the efficacy of EOs in downregulating capsule gene expression in MDR-KP. We employ a novel approach, utilizing specifically designed primers to quantify the downregulation of capsule genes in MDR-KP strains treated with essential oils. This study also incorporates microbiological assays and *in silico* analysis to provide a comprehensive understanding of the potential mechanisms involved. This approach offers a novel strategy to enhance antibiotic efficacy by targeting both bacterial survival and its ability to evade immune defenses.

## Materials and methods

### Essential oils

Ten different 100% pure essential oils were procured from the Medicinal and Aromatic Oils Unit at the National Research Center, Doki, Egypt: thyme, garlic, ginger, nigella, marjoram, moringa, tea tree, linseed, lemon, and sage.

### Ethical approval and sampling

This study adhered to the Declaration of Helsinki principles with informed consent obtained from all human participants. A total of 100 samples were collected between January and April 2024, comprising 50 bovine mastitic milk and 50 human samples including blood, urine, and swabs. The animal study protocol was approved by the Faculty of Veterinary Medicine, Zagazig University (ZU-IACUC/2/F/285/2023), and the human study protocol was approved by the Faculty of Medicine, Zagazig University (ZU-IRB/409/2024). Both protocols adhered to ARRIVE guidelines (PLoS Biol 8(6), e1000412, 2010).

### Isolation, identification and molecular confirmation of *Klebsiella pneumoniae* isolates

*Klebsiella* species isolates were obtained using the methods described by Garcia (2010). Bacterial cultures were grown on MacConkey agar and Eosin Methylene Blue then incubated at 37°C for 24 h. Molecular confirmation of *K. pneumoniae* was performed using a PCR assay targeting the 16S rRNA gene. DNA extraction was carried out using the QIAamp DNA Mini kit (Qiagen, Germany) followed by PCR amplification using specific primers (Table S2). A positive control (ATCC 13883) and a negative control (PCR mixture without DNA template) were included.

### Antimicrobial susceptibility testing of the bacterial isolates

#### Disk diffusion assay

*In vitro* determination of susceptibility pattern of *K. pneumoniae* isolates to various antimicrobials was conducted adopting the disk diffusion method (Bauer et al., 1966) using Mueller Hinton agar and standard antimicrobial disks including: amikacin (30 μg), norfloxacin (5 μg), tetracycline (10 μg), cefotaxime (5 μg), ceftriaxone (5 μg), ceftazidime (30 μg), chloramphenicol (30 μg), aztreonam (30 μg), sulfamethoxazole-trimethoprim (25 μg), cephalexin (30 μg), erythromycin (5 μg), and meropenem (10 μg). Plates were incubated at 37°C for 24 h, and inhibition zone diameters were measured. The results were interpreted according to the Clinical & Laboratory Standards Institute (CLSI, 2020) guidelines.

#### Agar well diffusion assay of herbal oils

The agar well diffusion assay was employed to assess the antibacterial activity of 10 distinct essential oils against isolated *Klebsiella* isolates. Briefly, bacteria were grown in nutrient broth, adjusted to a concentration of 1.5 × 10^8^ CFU/mL, and swabbed onto Muller-Hinton agar plates. After creating 7 mm-diameter wells in the agar, 100 μL of each essential oil, solubilized in 5% DMSO, was added. To account for the potential influence of DMSO on bacterial growth, control wells containing 5% DMSO alone were included. The plates were incubated at 37°C for 24 h. The diameter of inhibition zones surrounding the wells was measured. Inhibition zones less than 12 mm were considered indicative of no antibacterial activity based on previous studies of Durairaj et al. (2009) and El-Azzouny et al. (2018). Three replicates were performed for each sample, and the results were averaged.

#### Minimum inhibitory concentration and minimum bactericidal concentration determination

The experiment utilized 96 well plates (TPP, Switzerland) for the broth microdilution assay. The wells were injected with 1 × 10^5^ CFU of bacteria in a final volume of 0.2 mL after the effective extracts and chosen drugs were diluted twofold in Luria broth (LB) broth^®^ (Acumedia, Michigan, United States). Incubation took place for 24 h at 37°C. Following the guidelines of the Clinical and Laboratory Standards Institute (CLSI, 2020), MIC testing was conducted, with a concentration range of 0.062 to 1,024 μg/mL for each antimicrobial agent.

Subinhibitory concentration (*SIC*) refers to an antimicrobial agent’s concentration that is too low to completely inhibit microbial growth and replication, while the minimum inhibitory concentration (MIC) value is the lowest antimicrobial concentration that inhibits microbial growth. The minimum bactericidal concentration value (MBC) value was determined according to Khosravi and Malekan (2004) by subculturing colonies from wells exhibiting no visible growth onto fresh agar plates and incubating to assess bacterial viability.

#### Evaluation of synergistic effect

The checkerboard broth microdilution method was used to determine the synergy between antibiotics and plant extracts. Fractional inhibitory concentration (FIC) index was calculated to quantify the interaction. FIC value for each antimicrobial agent was calculated according to the formula described by van Vuuren and Viljoen (2011) and El-Demerdash et al. (2023d). The interactions were classified as being synergistic for ΣFIC values of ≤0.5, additive (≥0.5–1.0), indifferent (≥1.0 and ≤4.0) or antagonistic (ΣFIC > 4.0).

### Gas chromatography–mass spectrometry analysis

At the Central Laboratories Network of the National Research Centre in Cairo, Egypt, a mass spectrometer detector (5977A) and gas chromatograph (7890B) were equipped with an Agilent Technologies GC–MS system. Essential oils were analyzed directly and diluted with hexane at a ratio of 1:19 (v/v). The GC was outfitted with a DB-WAX column measuring 30 m × 250 μm in internal diameter and 0.25 μm in film thickness. The temperature program for the analysis began at 40°C for 2 min, then increased to 250°C at a rate of 7°C/min. It was held at 250°C for 8 min while injecting 1 μL of hydrogen without splitting using a carrier gas flow rate of 3.0 mL/min. The injector and detector were maintained at 250°C. Mass spectra were generated by electron ionization (EI) at 70 eV with a spectral range of m/z 40–550 and solvent delay 3.5 min. By comparing the fragmentation of the spectrum with data from the Wiley and NIST Mass Spectral Library, many constituents could be identified.

### *In silico* docking analysis

To investigate potential binding interactions, *in silico* docking studies were conducted using the extracted bioactive compounds and the crystal structures of FosAKP, *K. pneumoniae* K1 capsule, and OmpK36 (PDB IDs: 6C3U, 7W1E, 5o79). Crystal water molecules were eliminated, and hydrogen atoms were added to the protein structures prior to energy minimization using the MMFF94 force field. The 2D structures of the compounds were created using ChemBioDraw Ultra 14.0 and then energy minimized using the MMFF94 force field. Docking simulations were performed using LigandScout 2.0 (based on Autodock Vina), and the most favorable binding poses were chosen for analysis. The 3D and 2D binding modes were visualized using Biovia Discovery Studio Visualizer, and the results are summarized in Supplementary Table S1 (Al-Halbosy et al., 2023).

### Transmission electron microscopy assay

Bacterial samples were prepared for transmission electron microscopy (TEM) analysis following the methodology described by Amin et al. (2020). Briefly, *Klebsiella pneumoniae* cultures (both treated and control) were grown in nutrient broth for 24 h before centrifugation at 4,000 rpm for 10 min. The resulting pellet was washed with distilled water, fixed in 3% glutaraldehyde, and post-fixed in potassium permanganate for 5 min. Dehydration was achieved through a graded ethanol series (10–90%), culminating in absolute ethanol for 30 min. Samples were embedded in epoxy resin using an acetone gradient and ultrathin sections were cut and mounted on copper grids. Staining with uranyl acetate and lead citrate was followed by examination using a JEOL-JEM 1010 transmission electron microscope at 80 kV, located at the Regional Center for Mycology and Biotechnology (RCMB), Al-Azhar University, Egypt.

### Capsule gene detection and expression analysis

This section details the methods used for conventional PCR detection of capsule genes and their quantitative expression analysis using real-time PCR.

#### Conventional PCR for capsule gene detection

Supplementary Table S2 lists the primer sequences for target genes, along with expected amplicon sizes and annealing temperatures. A 25 μL reaction mixture was prepared containing 12.5 μL of DreamTaq Green PCR Master Mix (2X) from Thermo Scientific, 1 μL of each primer (20 pmol), 5.5 μL of nuclease-free water, and 5 μL of DNA template. The PCR was carried out using a 2,720 thermal cycler (Applied Biosystems) following the manufacturer’s instructions.

The PCR products were separated by electrophoresis on a 1% agarose gel (Applichem, Germany) in 1x TBE buffer at room temperature with a 5 V/cm gradient. For analysis, 10 μL of PCR products were loaded into each well. Fragment sizes were determined using Gelpilot 100 bp Plus DNA ladder (Qiagen) and the Generuler 100 bp ladder (Thermo Scientific).

#### Quantitative analysis of capsule gene expression

RNA was extracted from bacterial cultures using the QIAamp RNeasy Mini Kit (Qiagen). RNA concentration for each sample was measured using a NanoDrop Eight Spectrophotometer (Thermo Scientific). 16S rRNA was used as the internal control (housekeeping gene). Primer sequences are listed in Table 1. New primers targeting the *magA* and *wabG* genes were designed using Primer3 and FastPCR software. These primers were optimized for specificity and sensitivity using “touchdown PCR” and were validated through experimental testing.

**Table 1:** The utilized primers and their sequences of target genes for Syper green RT-PCR.

Genes	Primers (5′-3′)	Amplicon size	References
*16S rRNA*	F: ATT TGA AGA GGT TGC AAA CGA T R: TTC ACT CTG AAG TTT TCT TGT GTT C	130 bp	Turton et al. (2010)
*magA*	F: TGGCTTTATTGTTGCTGTGACA R: ACACTTCTCGTATTTGCGGC	230 bp	This study
*wabG*	F: AAGAGACCTTTGCCGCCTTA R: CCTTATCTTTGCCGACCACC	159 bp	This study
*wcaG*	F: GGTTGGKTCAGCAATCGTA R: ACTATTCCGCCAACTTTTGC	169 bp	Turton et al. (2010)
*rmpA*	F: AGAGTATTGGTTGACTGCAGGATTT R: AAACATCAAGCCATATCCATTGG	106 bp	Hartman et al. (2009)
*uge*	F: CTC TCA ACG GTC CAG TCG GC R: CCT GTA TGC CGC CAC CAA GA	288 bp	Nimnoi and Pongsilp (2022)

A 20 μL reaction mixture containing 10 μL of 2x HERA SYBR^®^ Green RT-qPCR Master Mix (Willowfort, UK), 1 μL of RT Enzyme Mix (20X), 0.5 μL of each primer (20 pmol), 3 μL of nuclease-free water, and 5 μL of RNA template was prepared. The reaction was carried out using a StepOne™ real-time PCR system (Applied Biosystems) following the manufacturer’s protocol. The program included an initial denaturation step at 94°C for 15 min, followed by 40 cycles of denaturation (94°C for 15 s), annealing (60°C for 30 s), and extension (72°C for 30 s). A final extension step at 72°C for 10 min concluded the reaction.

The StepOne^™^ software calculated CT values and generated amplification curves. The ΔΔCt method (Yuan et al., 2006) was used to compare the CT of each sample with the positive control group using the formula (2^−ΔΔCt^) to assess the variation in gene expression among the RNA samples. All molecular work was performed in the Biotechnology Unit, Animal Health Research Institute, Zagazig Branch, Egypt.

### Statistical analysis

Microsoft Excel (Microsoft Corporation, Redmond, WA, United States) was utilized to manipulate the data. Following the methodology of Razali and Wah (2011), a Shapiro–Wilk test was conducted to confirm normality. The One-way ANOVA (PROC ANOVA; Stokes et al., 2012) was employed to analyze the significant impacts of the treatments, with a significance level set at α = 0.05. The means ± SE of the results were then reported. In cases where a significant effect was observed. Tukey’s test was used to perform pairwise comparisons between means. A *p*-value threshold of less than 0.05 was established for determining statistical significance between means. The GraphPad Prism software 9.0 (GraphPad, United States) was used to create the figures.

## Results

### Prevalence rate

**Figure 1:**
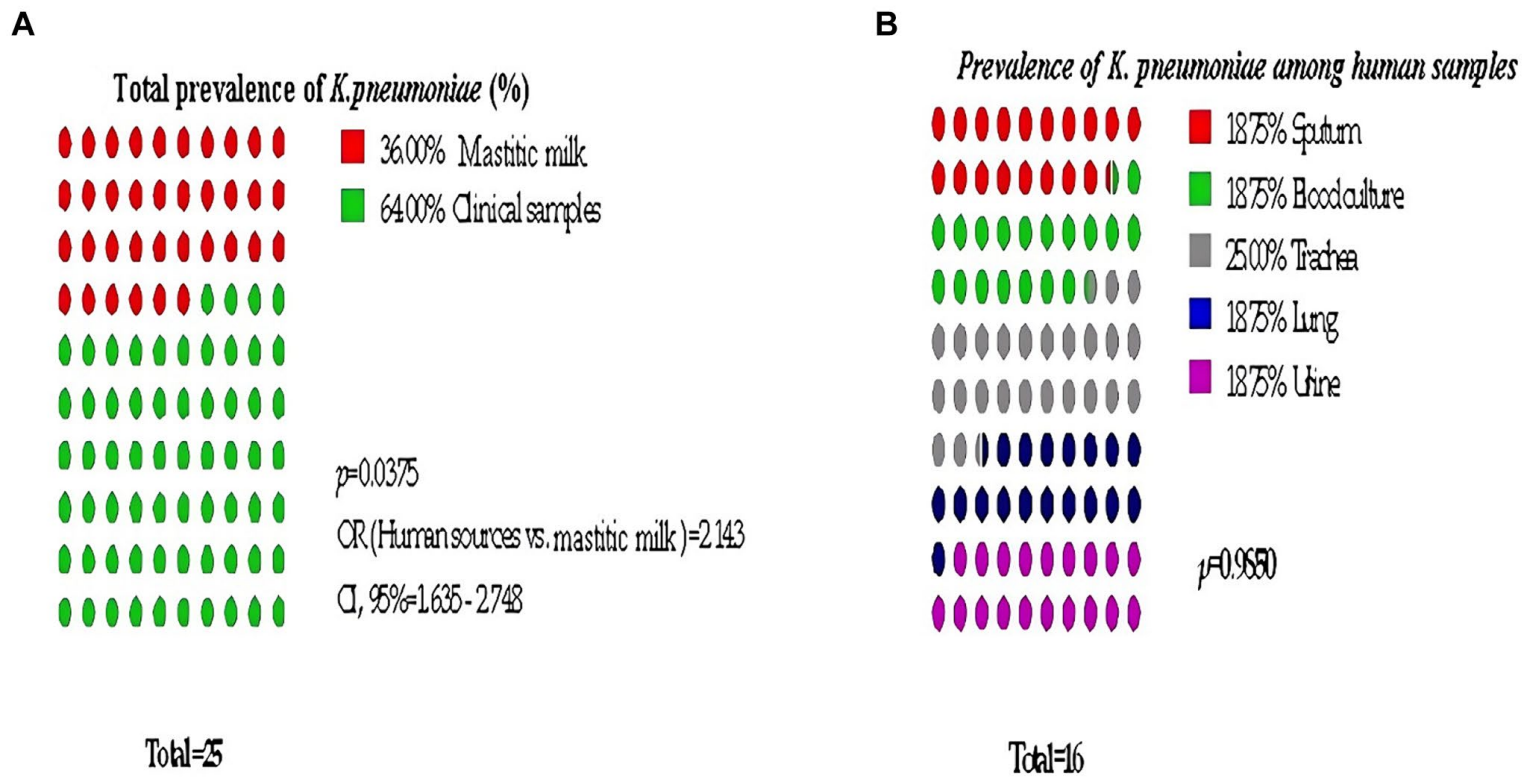
Significant prevalence of *K. pneumoniae* among examined samples. **(A)** Total prevalence, **(B)** Detailed Clinical samples prevalence.

Bacteriological examination revealed that 25 out of 100 samples were positive for *Klebsiella* spp. resulting in an overall prevalence rate of 25%. All of the isolates were identified as *Klebsiella pneumoniae* through biochemical and genotypical assays. The prevalence of *K. pneumoniae* in human sources was significantly higher than in mastitic milk, being approximately twice as high (OR = 2.143, 95% CL = 1.635–2.748, Figure 1).

### Antibiogram pattern of *Klebsiella pneumoniae* isolates

The antibiotic resistance rate for each source and the whole sets of isolates are represented in (Supplementary Figures S1, S2). The antimicrobial resistance pattern showed absolute resistance to cefotaxime followed by ceftriaxone and cephalexin (92%). More than half of the isolates exhibited resistance the rest tested antibiotics except amikacin. Notably, all 25 isolates were multidrug-resistant (MDR) i.e., resistant to three or more groups of antibiotics.

Regarding *K. pneumoniae* isolates from human sources, absolute resistance to cefotaxime and erythromycin was detected while the erythromycin resistance percentage for those from animal source (mastitic milk) was 55.5%.

In contrast, *K. pneumoniae* isolates from animal sources showed a low resistance rate to meropenem and chloramphenicol (27 and 55%, respectively), however, their percentages were high in isolates recovered from human sources (80 and 62%, respectively).

In total, *Klebsiella* isolates from human sources displayed a significant pattern of resistance, with almost all isolates showing high frequencies of resistance to more than seven drugs.

### Essential oil activity

**Figure 2:**
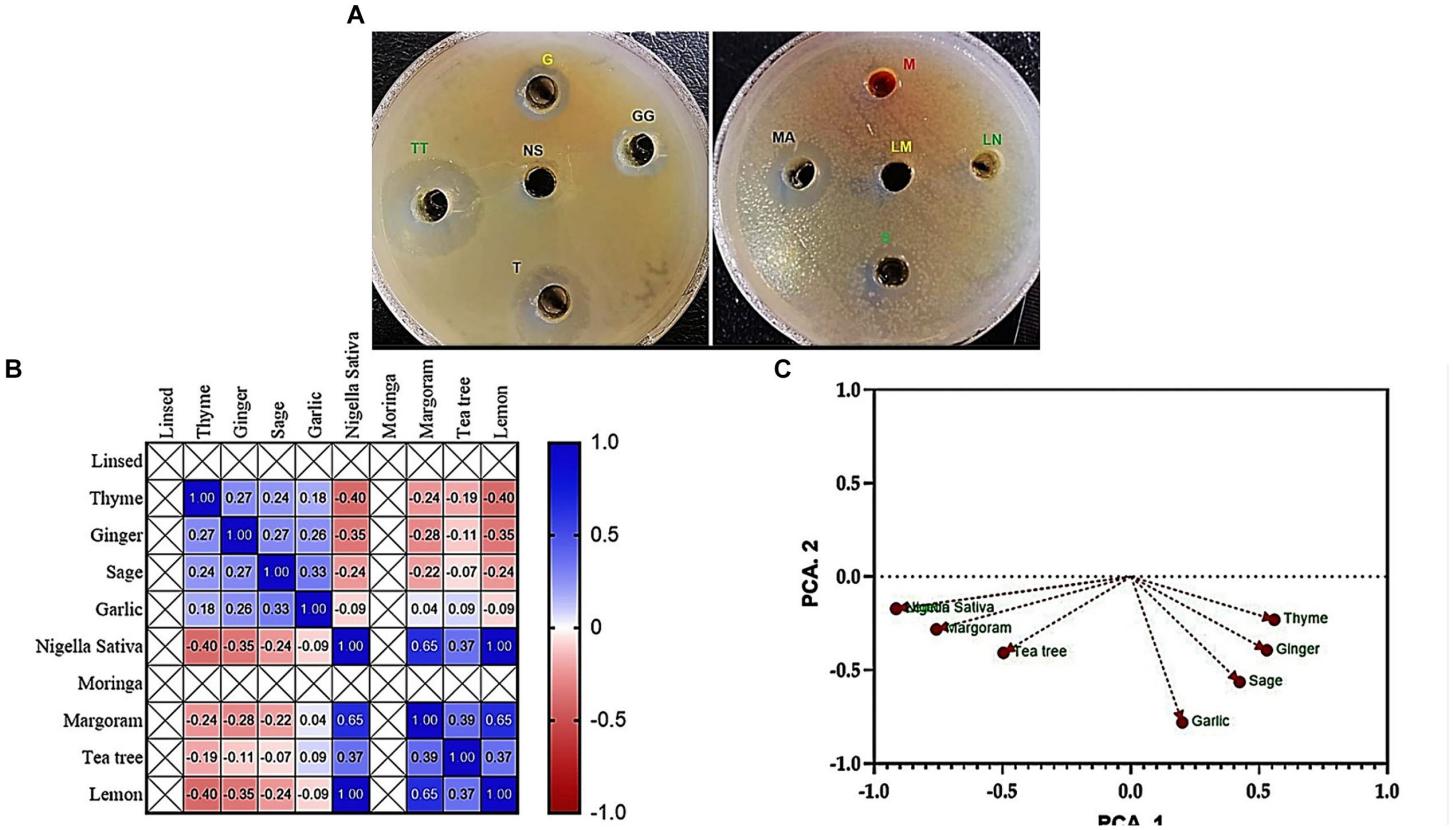
Antibacterial activity **(A)** Agar well diffusion assay shows the inhibition zones of *K. pneumoniae* (code No. KP11) caused by tested 10 oil extracts; TT, Tea tree; T, Thyme; MA, Margoram; LM, Lemon; NS, *Nigella sativa*; S, Sage; G, Garlic; GG, Ginger; M, Moringa; LN, Linseed. **(B)** Pearson’s correlation coefficient between inhibition zones (mm) of essential oils against MDR *K. pneumoniae*. **(C)** Principal component analysis (PCA) of inhibition zones (mm) of essential oils against MDR *K. pneumoniae.*

The results of the study showed that tea tree oil (TTO) exhibited the strongest antimicrobial properties followed by thyme among the 10 oils tested. Both oils showed inhibition zones ranging from 14 to 32 mm against the tested *Klebsiella* isolates. On other hand, moringa and linseed oils did not show any antibacterial activity against the tested pathogens (Figure 2A).

#### Pearson’s correlation

Pearson’s correlation coefficient (r) was performed to examine the relationship between the inhibition zones (mm) of essential oils and the prevalence of MDR *K. pneumoniae*. The results, presented in Figure 2B, showed a positive correlation between the inhibition zone values of tea tree oil and both thyme and margoram essential oils (*r* = 0.95 and 0.62, respectively; *p* < 0.05). Similarly, a highly positive correlation was observed between thyme and margoram (*r* = 0.61; *p* < 0.05). Conversely; a negative correlation coefficient was found between the other combinations of essential oils.

#### Principal component analysis

PCA was performed to identify associations between essential oil inhibition zones and the prevalence of MDR *K. pneumoniae*. The Varimax rotation method was used, and loadings greater than 0.50 or 0.60 were considered significant. Four principal components were extracted, explaining 87.98% of the total variance. The first component (17.77% variance) correlated with inhibition zones of tea tree, thyme, and marjoram oils. The second component (12.61% variance) was associated with sage and garlic oils, while the third component (9.51% variance) primarily represented ginger oil. The fourth component (8.50% variance) was associated with lemon oil (Table 2; Figure 2C).

**Table 2:** Varimax rotated principal component analysis (PCA) of inhibition zones (mm) of essential oils against MDR *K. pneumoniae* (bold loadings are statistically significant).

Essential oils	PC1	PC2	PC3	PC4
Lemon	0.549	−0.232	0.257	−0.736
Ginger	0.371	−0.507	0.628	0.280
Sage	−0.461	0.553	−0.227	0.257
Garlic	0.257	−0.748	−0.200	−0.003
Tea tree	−0.915	−0.166	0.250	0.091
Margoram	−0.748	−0.307	−0.187	−0.251
*Nigella sativa*	−0.459	−0.509	−0.469	−0.037
Thyme	−0.865	−0.223	0.374	−0.043
Eigenvalue	1.42	1.01	0.761	0.68
Proportion of variance	17.77%	12.61%	9.51%	8.50%
Cumulative proportion of variance	57.36%	69.97%	79.48%	87.98%

#### Minimum inhibitory concentration and synergy with amikacin

Tea tree oil displayed the most potent antibacterial activity against *K. pneumoniae* isolates, with MICs ranging from 2 to 32 μg/mL (Table 3; Supplementary Figure S3). Thyme oil also exhibited inhibitory effects (MICs: 0.5–256 μg/mL). Amikacin, a conventional antibiotic, showed good activity with MICs of 2–4 μg/mL.

**Table 3:** SIC, MIC, and MBC of tea tree oil, thyme oil and amikacin against *Klebsiella* isolates.

Isolate no.	Code no.	Concentration of SIC, MIC, MBC (μg/ml)								
		Tea tree oil extract			Thyme oil extract			Amikacin		
		SIC	MIC	MBC	SIC	MIC	MBC	SIC	MIC	MBC
1	2K	8	16	32	64	128	256	1	2	4
2	3K	1	2	4	128	256	512	1	2	4
3	10K	8	16	32	32	64	128	2	4	8
4	14K	8	16	32	0.25	0.5	1.0	2	4	8
5	15K	16	32	64	0.25	0.5	1.0	1	2	4
6	16K	8	16	32	2	4	8	1	2	4
7	17K	4	8	16	16	32	64	2	4	8
8	19K	8	16	32	32	64	128	1	2	4

Further investigations explored the synergistic potential of these essential oils with amikacin. Both tea tree and thyme oil combinations displayed synergistic effects against most isolates, with tea tree oil exhibiting a stronger synergy (FIC index: 0.1325–0.53) compared to thyme oil (FIC index: 0.15–0.53). Notably, the synergistic interaction was more pronounced for resistant isolates. Overall, these findings suggest that combining essential oils with amikacin could be a promising strategy to combat *K. pneumoniae* infections (Tables 4, 5).

**Table 4:** MIC of tea tree oil and amikacin alone and in combination and FIC index against *Klebsiella* isolates by the checkerboard method.

Isolate no.	Code no.	MIC of tea tree oil	MIC of amikacin	MIC of tea tree oil in combination	MIC of amikacin in combination	FIC of tea tree oil	FIC of amikacin	Ʃ FIC	Interpretation
1	2K	16	2	4	0.06	0.25	0.03	0.28	Synergistic
2	3K	2	2	0.5	0.015	0.25	0.0075	0.257	Synergistic
3	10K	16	4	4	0.125	0.25	0.03	0.28	Synergistic
4	14K	16	4	2	0.03	0.125	0.075	0.1325	Synergistic
5	15K	32	2	4	0.06	0.125	0.03	0.155	Synergistic
6	16K	16	2	0.5	0.5	0.03	0.5	0.53	Partially synergistic
7	17K	8	4	1	0.125	0.125	0.03	0.155	Synergistic
8	19K	16	2	4	0.06	0.25	0.03	0.28	Synergistic

**Table 5:** MIC of thyme oil and amikacin alone and in combination and FIC index against *Klebsiella* isolates by the checkerboard method.

Isolate no.	Code no.	MIC of thyme Oil	MIC of amikacin	MIC of thyme oil in combination	MIC of amikacin in combination	FIC of thyme oil	FIC of amikacin	Ʃ FIC	Interpretation
1	2K	128	2	64	0.06	0.5	0.03	0.53	Partially synergistic
2	3K	256	2	128	0.015	0.5	0.0075	0.507	Synergistic
3	10K	64	4	8	0.125	0.125	0.03	0.155	Synergistic
4	14K	0.5	4	0.125	0.03	0.25	0.0075	0.257	Synergistic
5	15K	0.5	2	0.06	0.06	0.12	0.03	0.15	Synergistic
6	16K	4	2	2	1	0.5	0.05	0.55	Partially synergistic
7	17K	32	4	8	0.125	0.25	0.03	0.28	Synergistic
8	19K	64	2	16	0.06	0.25	0.03	0.28	Synergistic

### Characterization of compounds present in the oily extracts (thyme and tea tree oils) by using GC–MS

Supplementary Tables S3, S4 and Supplementary Figures S4, S5 detail the characterization of compounds in the effective extracts. GC–MS analysis of tea tree oil identified seven bioactive compounds, with Terpinene-4-ol exhibiting the highest peak area. Other identified compounds included P-cymene, Alpha-Terpineol, Beta-pinene, Beta-myrcene, Limonene, and Farnesol. Similarly, thyme oil analysis revealed 10 chemical compounds, with Thymol being the principal bioactive component followed by p-cymene, Gamma terpinene, Linalool, and Eugenol.

### Docking data

**Figure 3:**
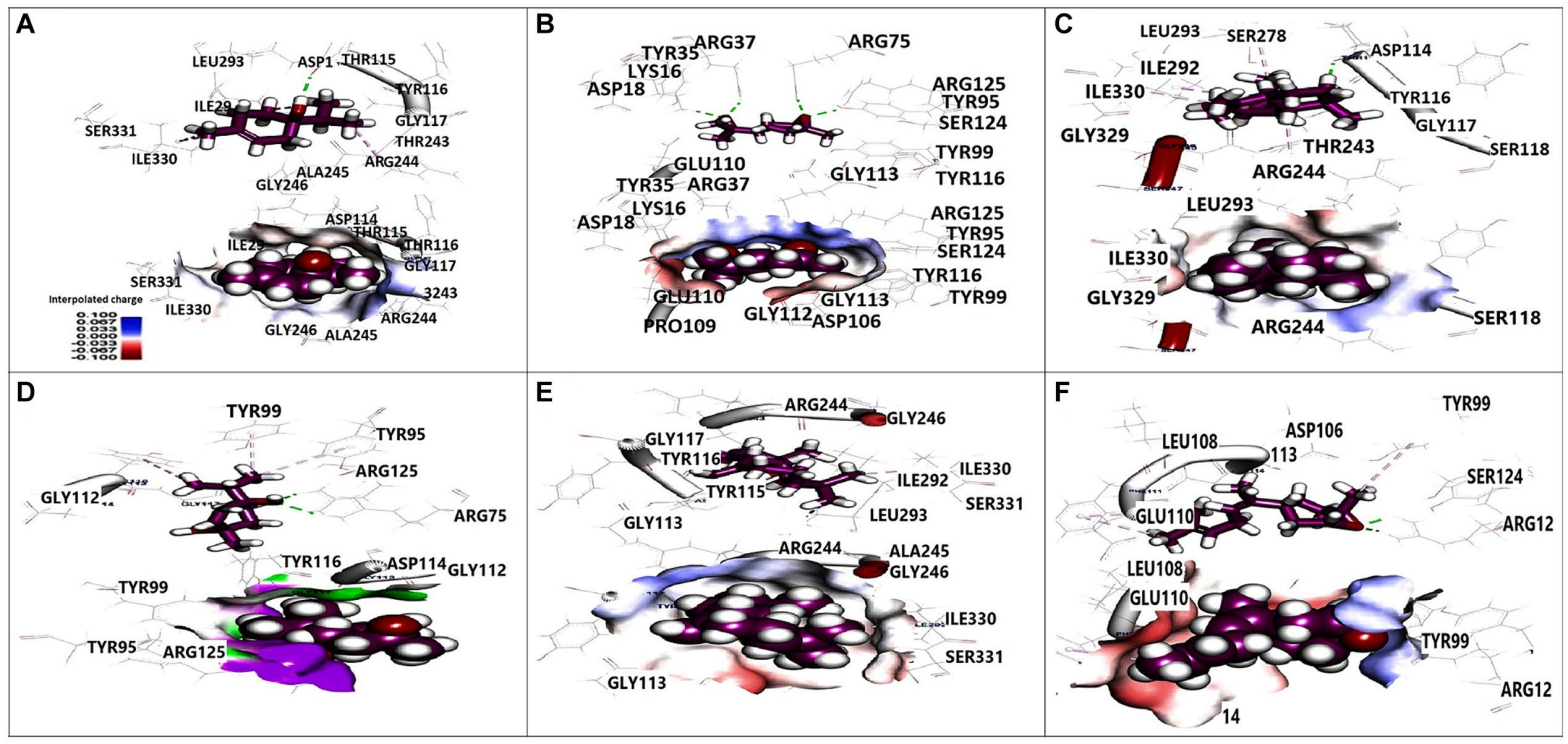
3D orientation and surface mapping of active components against OmpK36 target site. **(A)** Terpinen-4-ol, **(B)** Farnesol, **(C)** (+) spathulenol, **(D)** 1,4-dihydroxy-p-menth-2-ene, **(E)** (−) spathulenol, **(F)** trans-Z-. alpha. -Bisabolene epoxide.

**Figure 4:**
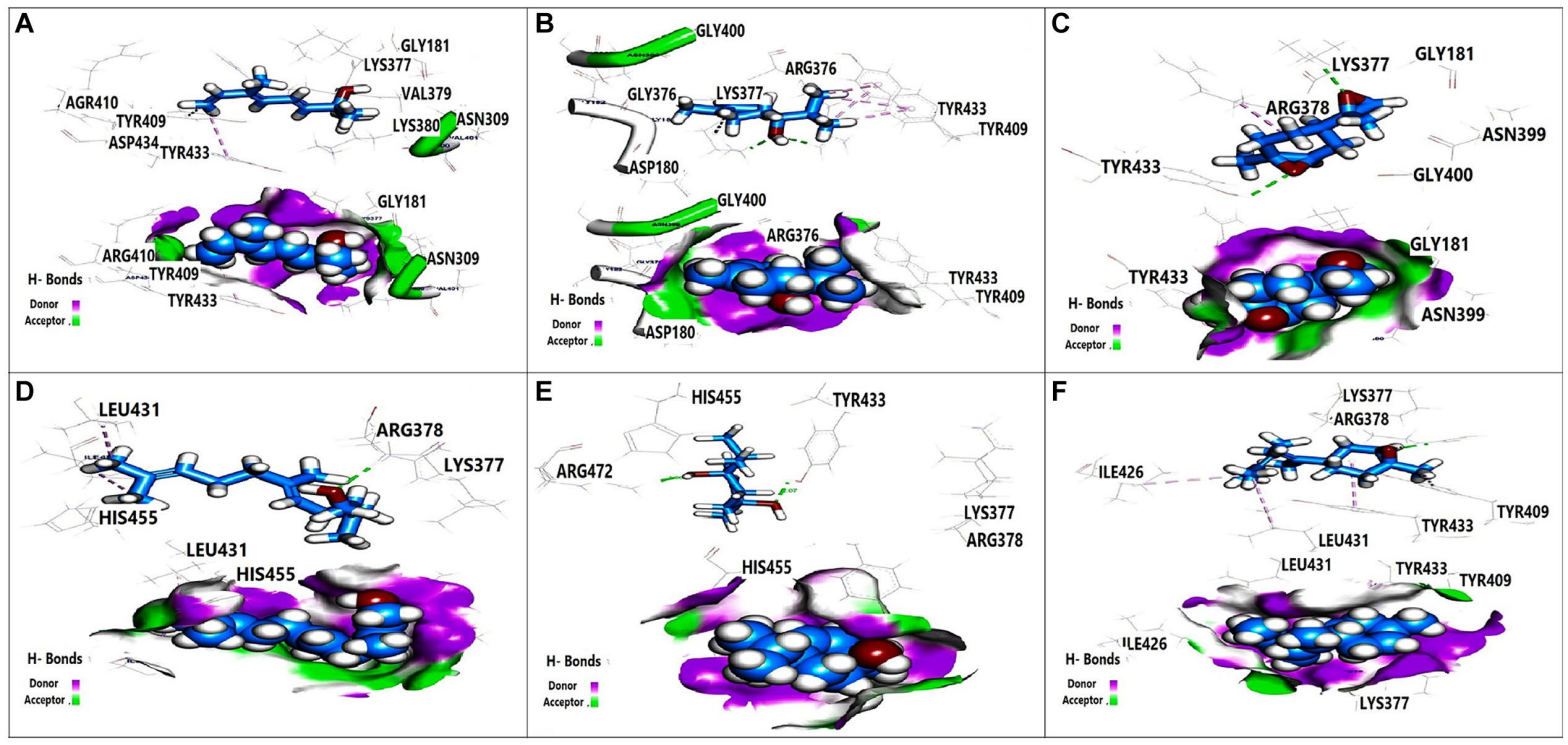
3D orientation and surface mapping of active components against *K. pneumoniae* K1 target site. **(A)** 2,6-Dimethyl-3,5,7-octatriene-2, **(B)** Terpinen-4-ol, **(C)** 7-Oxabicyclo, **(D)** Farnesol, **(E)** 1,4-dihydroxy-p-menth-2-ene, **(F)** trans-Z alpha. -Bisabolene epoxide.

**Figure 5:**
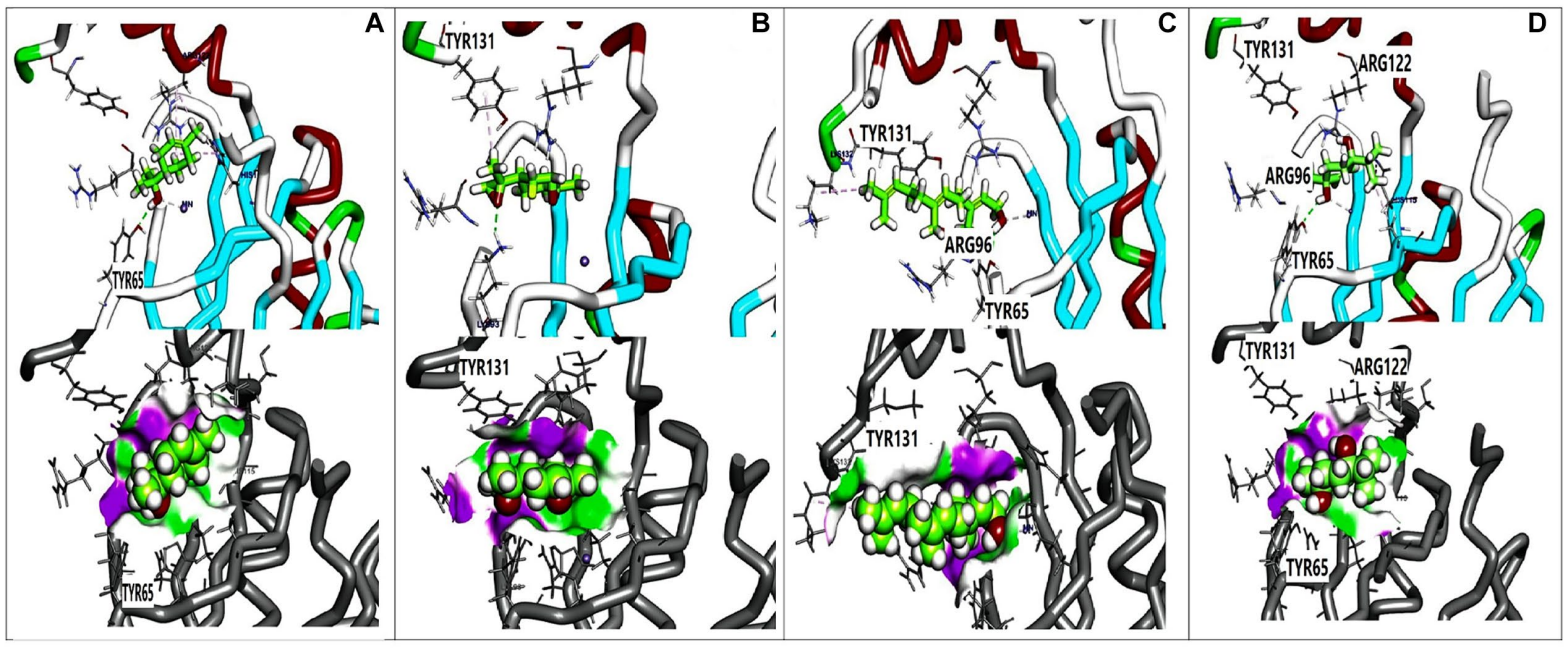
3D orientation and surface mapping of active components against FosAKP target site. **(A)** Terpinen-4-ol **(B)** *7-oxabicyclo* **(C)** Farnesol **(D)** 1,4-dihydroxy-p-menth-2-ene.

Molecular docking analysis was performed to investigate the potential binding interactions of identified bioactive compounds with target proteins in *K. pneumoniae* (OmpK36, K1 capsule, and FosAKP). The results revealed favorable binding energies for several compounds with each target protein. For example, Terpinen-4-ol exhibited strong binding affinity for all three targets (OmpK36: −6.25 kcal/mol; K1 capsule: −5.81 kcal/mol; FosAKP: −5.94 kcal/mol). Similarly, other identified compounds like 1,4-dihydroxy-p-menth-2-ene, 7-Oxabicyclo [4.1.0] heptane, and trans-Z-.α.-Bisabolene epoxide also demonstrated promising binding interactions (Figures 3–5).

### Transmission electron microscopy

**Figure 6:**
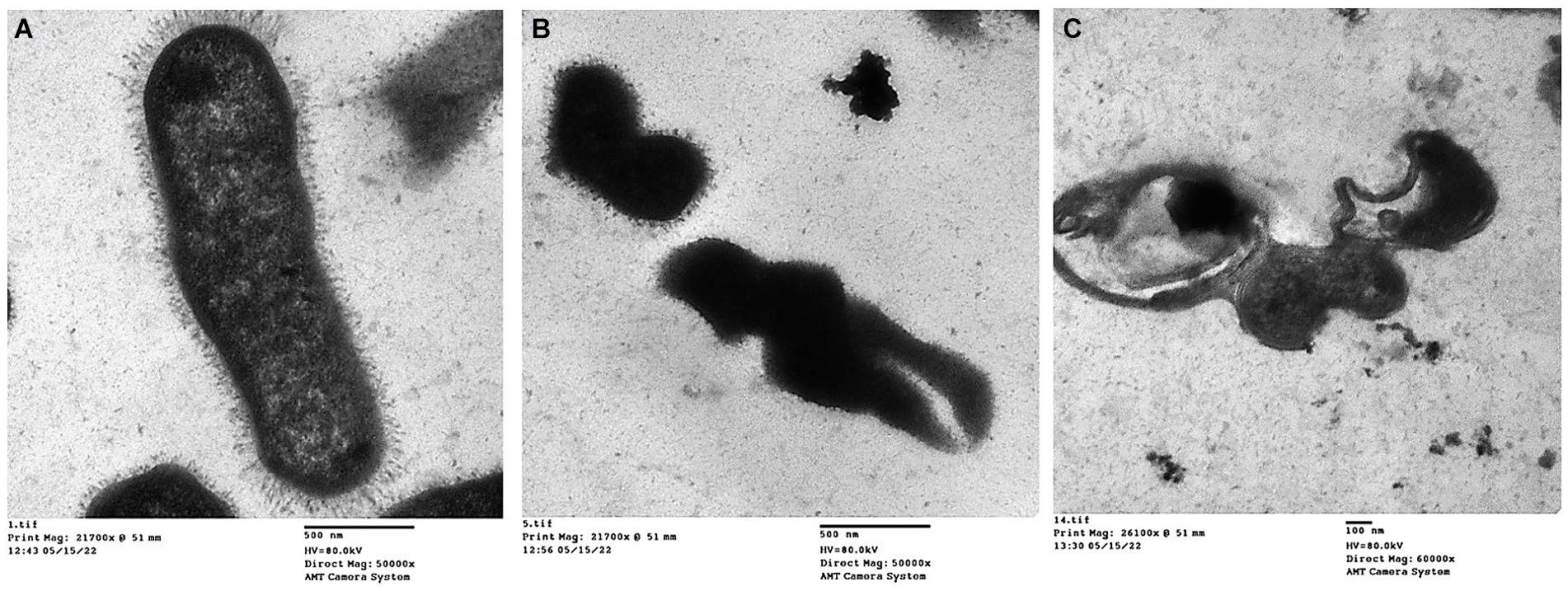
Transmission electron micrographs of *K. pneumoniae* cells; **(A)** Untreated *K. pneumoniae* cells; **(B)** *K. pneumoniae* cells treated with thyme oil and amikacin, and **(C)** *K. pneumoniae* cells treated with tea tree oil and amikacin.

Transmission electron micrographs revealed distinct morphological changes in *K. pneumoniae* cells following treatment with essential oils and amikacin. Untreated cells (Figure 6A) exhibited a normal rod-shaped morphology with a characteristically rough (rugose) and rigid cell surface. In contrast, cells treated with the combination of thyme oil and amikacin (Figure 6B) displayed significant alterations, including a crumpled and shrunken cell surface with irregular shapes. Notably, some cells exhibited outward openings and cleavages in the cell wall. Treatment with tea tree oil and amikacin (Figure 6C) resulted in even more severe damage, characterized by complete disruption of the cell envelope and leakage of cytoplasmic contents. These lysed cells appeared devoid of internal structures and possessed collapsed and fragmented cell walls.

### Detection of capsule genes by conventional PCR

The detection of capsule genes in *Klebsiella* isolates was confirmed by the PCR. Five different primers which produced 169-bp, 516-bp, 1,282-bp, 534-bp, and 683-bp PCR products, enabled elucidating the presence of capsule genes in the genomes of *Klebsiella* isolates. This was obvious when the *Klebsiella* isolates compared to a positive control (Lane +C) as in Supplementary Figure S6.

### Transcriptional analysis of capsule expression genes of *Klebsiella pneumoniae*

**Figure 7:**
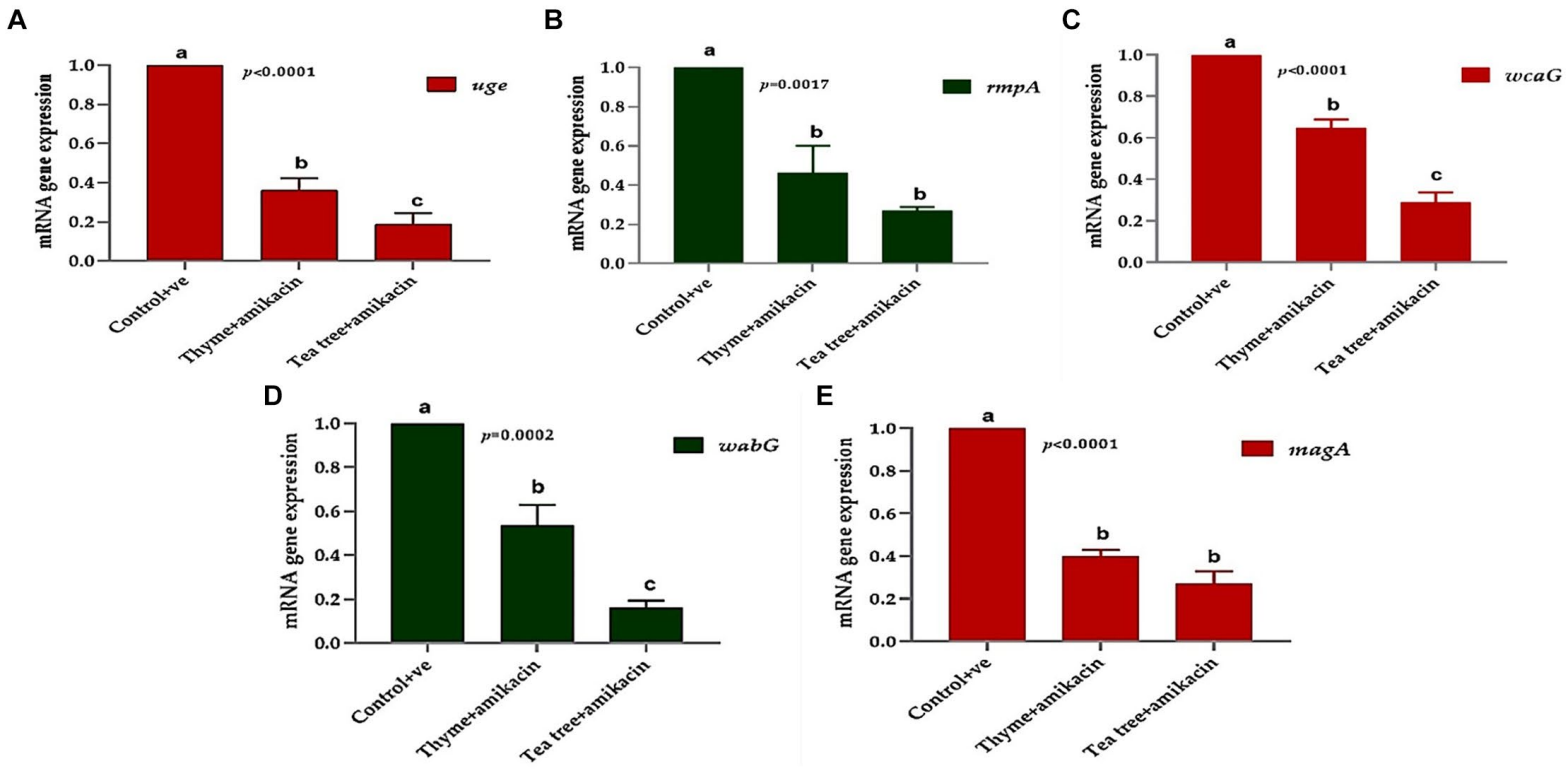
The relative mRNA expression levels of genes related to capsule formation through tested *K. pneumoniae* isolates before and after treatments; **(A)** *uge*, **(B)** *rmpA*, **(C)** *wcaG*, **(D)** *wabG*, and **(E)** *magA.*

The relative expression of five virulence genes (*wcaG, rmpA, magA, uge*, and *wabG*) which are required for capsule formation, were measured in *Klebsiella* isolates treated with tea tree oil plus amikacin and thyme oil plus amikacin as illustrated in Figure 7. The 16 s rRNA housekeeping gene was used for qRT-PCR normalization.

Using RT-PCR, the amounts of the tested virulence gene products before and after treatment with thyme oil combined with amikacin can be compared. The fold change in the *magA* gene before treatment was 1 and after treatment, it was downregulated and reached 0.341 in the tested isolates.

The fold change in the *wabG* gene after treatment ranged from 0.353 to 0.683. The same downregulation occurred in the *wcaG* gene with a fold change of 0.562. Additionally, the *rmpA* and *uge* genes were downregulated with fold change ranges of 0.275–0.732 and 0.244–0.444, respectively.

Regarding the tea tree + amikacin-treated group, strong downregulation of the same virulence genes was detected. The *magA* gene exhibited fold change ranging from 0.15 to 0.34, the *wabG* gene had fold change ranges of 0.100–0.213, *wcaG* fold changes of 0.194–0.356, *rmpA* fold changes of 0.233–0.301 and *uge* gene had fold change ranging from 0.1060 to 0.299.

Overall, the treatments significantly affected the studied genes; the expression of *uge, wcaG,* and *wabG* was significantly downregulated (*p* < 0.05) in the tea tree + amikacin groups compared to thyme + amikacin treated groups (Figures 7A,C,D). Meanwhile, non-significant differences (*p* > 0.05) were observed between the aforementioned two groups for the expression of *rmpA* and *magA* genes (Figures 7B,E).

## Discussion

Today, *K. pneumoniae* is considered the most common cause of hospital-acquired pneumonia in most countries worldwide (Anes et al., 2017). The emergence of multidrug resistant bacteria (MDR) isolates of *K. pneumoniae* is a global issue (Ripabelli et al., 2018). Numerous studies have isolated MDR *K. pneumoniae* from various animals and humans (Yang et al., 2019; El-Demerdash et al., 2023b). However, the correlation between capsule formation and antibiotic resistance in *K. pneumoniae* is not fully understood. Therefore, this study was conducted to investigate the prevalence of MDR *Klebsiella* isolates in animals and human in Egypt, as well as to evaluate possible solutions to overcome this antibiotic resistance and their ability to form capsules by using different herbal extracts.

In this study, *K. pneumoniae* was recovered from 25 out of 100 samples, yielding an overall prevalence of 25%. This finding is comparable to the prevalence range reported in previous studies on *Klebsiella* colonization in Western countries (5–35%) but notably higher than the reported rates in Europe and North America (5 and 3%, respectively) (Chang et al., 2021; Salari et al., 2023).

However, direct comparisons between regions can be misleading due to variations in sample types, study populations, and methodological approaches. For instance, while the overall prevalence in Africa is reported as 45% (Salari et al., 2023), significant heterogeneity exists within the continent. To provide a more accurate context, future studies should focus on specific regional comparisons, considering factors such as healthcare settings, antibiotic usage, and socioeconomic conditions.

The abuse and misuse of antimicrobial medicines are leading to the emergence of resistance to these agents as a global crisis in the management of infections associated with *K. pneumoniae* (El-Demerdash et al., 2018; Ali et al., 2023; Ndlovu et al., 2023). This study highlights a concerning case of highly diversified antibiotic resistance. Data from the Chinese Antimicrobial Resistance Surveillance System (CARSS) indicates that *K. pneumoniae* is the second most common (20.2%) gram-negative infection among isolated bacteria.

The present study also investigated the low sensitivity of *K. pneumoniae* isolates to tetracycline. The low sensitivity of tetracycline is due to mutations in the chromosomes in the outer membrane of bacteria leading to a decrease in tetracycline penetration into the cell (Grossman, 2016).

Disk diffusion susceptibility testing revealed that all isolated *K. pneumoniae* strains (100%) were multidrug-resistant (MDR). This finding aligns with previous reports by (Del Prete et al., 2019; Ferreira et al., 2019) who also observed a high prevalence of MDR *K. pneumoniae*. Notably, the disk diffusion method likely identified high resistance to key β-lactam antibiotics such as cefotaxime and ceftriaxone, which are commonly used for treating *Klebsiella* infections. This resistance contributes significantly to the designation of MDR.

According to other sources, the MDR pattern may be attributed to the excessive use of antibiotics in Egyptian veterinary and human medicine, as well as the horizontal or vertical transmission of plasmids carrying antimicrobial resistance genes among different bacterial pathogens or between animals and humans (Witte, 1998; El Damaty et al., 2023; El-Demerdash et al., 2023a, 2023c; Saad et al., 2024). On the other hand, the best susceptibility (52%) was observed for amikacin which is an aminoglycoside antibiotic that prevents bacteria from synthesizing proteins by binding to the 30S ribosomal subunit mRNA and causing reading mistakes. Amikacin is also highly resistant to modification by bacterial enzymes leading many bacteria to be sensitive to this antibiotic (Ramirez and Tolmasky, 2017).

In the search for alternative antimicrobial agents, several studies have shown the potential of essential oils in fighting MDR bacteria. Terpenes and terpenoids are common volatile low molecular weight chemicals found in complex hydrophobic liquids known as essential oils (EOs). They are extracted from plants through solvent extraction, mechanical expression, or distillation. Previous studies have demonstrated the antibacterial properties of essential oils and their constituents against gastrointestinal and other infections (Millezi et al., 2016; El-Demerdash et al., 2023d). Building on this evidence, the present study evaluated the effectiveness of 10 essential oils against 25 *Klebsiella* isolates.

The antimicrobial activities of essential oils are attributed to their ability to penetrate microbial cells, causing structural and functional changes due to their hydrophobic nature. This disruption of the cytoplasmic membrane leads to cell lysis and the release of intracellular compounds (Lopez-Romero et al., 2015). Moreover, the diverse mechanisms of action and functional diversity of essential oils increase microbial sensitivity (Bakkali et al., 2008; Nazzaro et al., 2013). Among the tested plant extracts, tea tree and thyme oils exhibited high antimicrobial activities. Therefore, this study recommends the use of tea tree and thyme oils as antimicrobial agents and chemical preservatives due to their relatively lower toxicity and side effects (Santurio et al., 2014). Additionally, the multi-component nature of plant extracts makes it more difficult for bacteria to develop resistance compared to commonly used antibiotics, which have a single target site (El-Azzouny et al., 2018).

Without a doubt, the antibacterial action of essential oils is determined by their chemical structure which can vary depending on factors such as weather, soil type, and geographic location (Dardona, 2014; Dardona and Al-Hindi, 2019). Therefore, it is crucial to understand the chemical composition of essential oils in order to connect it to their antibacterial properties GC/MS was utilized to conduct chemical profiling of the oils extracted from tea trees and thyme.

This study uniquely investigates the efficacy of tea tree oil against MDR-KP strains, a significant contribution as it opens new avenues for alternative therapeutic strategies to address the growing challenge of antibiotic resistance. GC-mass analysis revealed the presence of various bioactive compounds in the effective tea tree oil, potentially contributing to the observed efficacy through a synergistic mechanism. The most abundant component, terpinen-4-ol, previously linked to biofilm inhibition in *S. aureus* (Cordeiro et al., 2020), along with limonene, another component with documented antibacterial activity (Slade et al., 2009; Matsuo et al., 2011), might contribute to the observed synergistic mechanism.

Similar to tea tree oil, thyme extract, analyzed using GC–MS, revealed a rich composition of bioactive compounds. Thymol, the primary component, is recognized for its broad spectrum of antimicrobial activity (Kowalczyk et al., 2020; Khan et al., 2021). Studies suggest that thymol disrupts bacterial membranes through interactions with membrane proteins and potentially by altering ATP levels (Tiwari et al., 2009). Additionally, thyme oil contains carvacrol, another well-documented antibacterial compound, known to inhibit flagella formation in bacteria (Burt et al., 2007). These findings highlight the potential mechanisms by which thyme extract might exert its antibacterial effects against MDR-KP strains.

However, a major limitation of essential oils is the high dosage required to combat resistant microorganisms, hindering their therapeutic application (Hyldgaard et al., 2012). This study investigated the potential of combining antibiotics with well-selected plant extracts, like tea tree and thyme oils, to address this challenge. Our findings demonstrate that this combination strategy offers several advantages: (i) overcoming resistance, (ii) reducing effective antibiotic dosages (leading to lower costs and minimized side effects), and (iii) broadening the spectrum of efficacy against MDR-KP strains (Hyldgaard et al., 2012; Purkait et al., 2020). Notably, the Fractional Inhibitory Concentration (FIC) index calculations revealed a strong synergistic effect between the antibiotics and herbal extracts, suggesting a combined action that is significantly more potent than their individual effects.

The observed synergy might be attributed to the ability of herbal extracts, particularly tea tree and thyme oil, to enhance the penetration of antibiotics, like amikacin, through the outer membrane of MDR-*KP. In silico* docking analysis further supported this hypothesis by revealing potential interactions between identified bioactive compounds within the extracts and key target proteins in *K. pneumoniae*. These targets included OmpK36, a porin crucial for outer membrane permeability, the K1 capsule polysaccharide essential for bacterial virulence, and FosAKP, an enzyme vital for lipopolysaccharide (LPS) biosynthesis.

Several compounds displayed promising binding affinities for OmpK36, suggesting they might disrupt its function. Terpinen-4-ol, with the strongest binding energy, interacted with hydrophobic residues and formed a hydrogen bond with Asp114 (Figure 3A). Similar interactions were observed with other compounds (Figures 3C,F). Disruption of OmpK36 could hinder nutrient uptake and antibiotic penetration by *K. pneumoniae*.

Docking analysis revealed favorable interactions between essential oil compounds and the K1 capsule biosynthesis machinery. Terpinen-4-ol once again demonstrated notable binding affinity for the K1 capsule target site (Figure 4B), suggesting potential interference with K1 capsule assembly. This could weaken the bacterial cell envelope and enhance antibiotic efficacy.

The essential oil compounds also showed binding potential for FosAKP.Terpinen-4-ol and 1,4-dihydroxy-p-menth-2-ene displayed favorable binding energies with interactions involving hydrophobic residues, hydrogen bonds, and metal ion interactions (Figures 5A,D). This suggests a potential mechanism for inhibiting LPS synthesis, another critical component of the bacterial outer membrane.

Overall, the docking analysis results provide compelling evidence for the potential mechanisms by which essential oil components might exert their antibacterial effects against *K. pneumoniae*. The observed interactions with key target proteins like OmpK36, K1 capsule components, and FosAKP suggest that these compounds might disrupt essential cellular processes, leading to membrane permeability alterations, reduced virulence, and ultimately, bacterial cell death. However, it is important to acknowledge that *in silico* results need to be validated through wet lab experiments to confirm the predicted binding modes and their functional consequences.

One of *K. pneumoniae*’s most significant virulence factors in terms of infection-causing capacity is capsular polysaccharide (CPS). Polymorphonuclear cells seen in CPS essentially form the pathogen’s outer coating and provide resistance to phagocytosis. By lowering the quantity of C3 on the bacteria and functioning as a barrier to prevent contact between macrophage receptors and their ligands on the bacterial surface, CPS reduces the connection between bacterial cells (Opoku-Temeng et al., 2019).

The transmission electron microscopy (TEM) results showed detailed cell damage of the *Klebsiella* isolate after treatment with a combination of essential oil and amikacin.

The TEM revealed that the size of capsules in treated cells, by both agents, was profoundly significantly reduced compared to that of control untreated cells. Overall, it has been documented that plant-derived compounds may be a promising resource for developing novel therapeutic approaches targeting bacterial capsule production (Upadhyay et al., 2014). Additionally, Dumlupinar et al. (2020) reported that the multicomponent structures of essential oils particularly tea tree oil reduce the potential for biosynthesis of bacterial capsular polysaccharides among virulent *K. pneumoniae* isolates.

In the current study, the use of essential oils (tea tree oil and thyme oil) in combination with amikacin downregulated the expression of genes responsible for capsule synthesis in *Klebsiella pneumoniae.*

The most important genes involved in capsule formation are *magA, rmpA, uge, wcaG* and *wabG*. The *magA* gene (muco viscosity associated gene A) was originally identified through transposon mutagenesis screening (Costa, 2019). *MagA* was identified based on its role in mucoviscosity, resistance to serum killing and phagocytosis, and virulence in mice (Fang et al., 2004). Mucoviscosity is indirectly related to *magA* because of its essential role in capsule production. While mucoviscosity might be mediated by capsule expression-promoting regulators such as *rmpA* (Hsu et al., 2011).

The production of capsule polysaccharides is regulated by the *rmp*A gene which has been identified in three forms: *rmp*A2 on a plasmid, *rmp*A located chromosomally, and *rmp*A on a plasmid (Shon et al., 2013). Hypercapsule production controlled by *rmp*A, inhibits opsonophagocytosis and phagocytosis of *K. pneumoniae* by the host’s immune cells. This also suppresses opsonization and lysis caused by complement. Ikeda et al. (2018) and Khan et al. (2021) have demonstrated that *rmp*A regulation is linked to *K. pneumoniae*’s ability to evade immune responses, emphasizing the critical role of the *rmp*A gene in the progression of infection.

Conversely, most isolates from urine include the *uge* gene (uridine diphosphate galacturonate-4epimerase). Studies have shown that *K. pneumoniae* strains lacking the *uge* gene are less pathogenic and unable to cause sepsis, pneumonia, or UTI. In experimental settings where urinary infections are induced, mutations in the *uge* gene have been found to decrease *K. pneumoniae*’s ability to colonize (Izquierdo et al., 2003; Ballén et al., 2021).

Similarly, Shu et al. (2009) have demonstrated that the *wcaG* virulence gene (GU325787) located in the transferable regions of the chromosome is responsible for *K. pneumoniae* capsule biosynthesis. It is needed for the conversion of mannose to fucose, which may enhance the ability of bacteria to evade phagocytosis by macrophages.

Additionally, almost all clinical isolates possess the significant gene *wab*G. Most research indicates that 88–100% of *K. pneumoniae* isolates harbor *wab*G, while one study only found *wab*G in 5.3% of isolates (Izquierdo et al., 2003). Furthermore, Jung et al. (2013) observed that in intraperitoneal, pneumonic, and UTI rodent infection models, *K. pneumoniae* strains lacking this gene are attenuated and unable to produce the Lipopolysaccharide (LPS) outer core or maintain capsular antigen.

qRT-PCR analysis revealed a dampening effect of the essential oil combinations on the fold change of the previously mentioned capsule gene expression. Notably, the tea tree oil combination exhibited a significantly stronger influence compared to thyme. These results suggest that tea tree oil might act as a more potent anti-virulence and antibiotic-resistance modifying agent when compared to thyme. Furthermore, the observed dampening effect on capsule gene expression by the essential oil combinations highlights a potentially novel mechanism by which they exert their antibacterial activity against *K. pneumoniae*.

These results indicate that essential oils were able to down-regulate the expression of the investigated genes which signifies their antagonistic properties against *Klebsiella*. Decreasing *Klebsiella* virulence through the synergistic effect of essential oils and antibiotics would reduce the pathogen’s ability to cause infection. This is an important concept in the era of antibiotic resistance.

In conclusion, this study highlights the promising potential of combining conventional antibiotics with essential oils derived from medicinal plants (such as tea tree and thyme) for developing novel antimicrobial therapies and mitigating drug resistance. The synergistic activity observed between the tested antibiotics and essential oils opens up new treatment strategies against various microbial infections and provides hope for combating the growing threat of antibiotic resistance.

However, it is important to note that these findings are based on *in vitro* experiments. Further *in vivo* studies are necessary to confirm the observed synergy and evaluate the efficacy and safety of these combinations in a more complex biological system Additionally, understanding the molecular mechanisms underlying this synergistic interaction is crucial for the development of effective antibacterial drugs derived from medicinal plants.

## Data Availability

The original contributions presented in the study are included in the article/Supplementary material, further inquiries can be directed to the corresponding authors.
