# Supplementary material for: Essential oils as capsule disruptors: enhancing antibiotic efficacy against multidrug-resistant Klebsiella pneumoniae
Source: Front Microbiol. 2024 Aug 30;15:1467460. doi: 10.3389/fmicb.2024.1467460 (PMC11392748; doi:10.3389/fmicb.2024.1467460)
Supplement: Supplementary file 1 [file Data_Sheet_1.pdf]

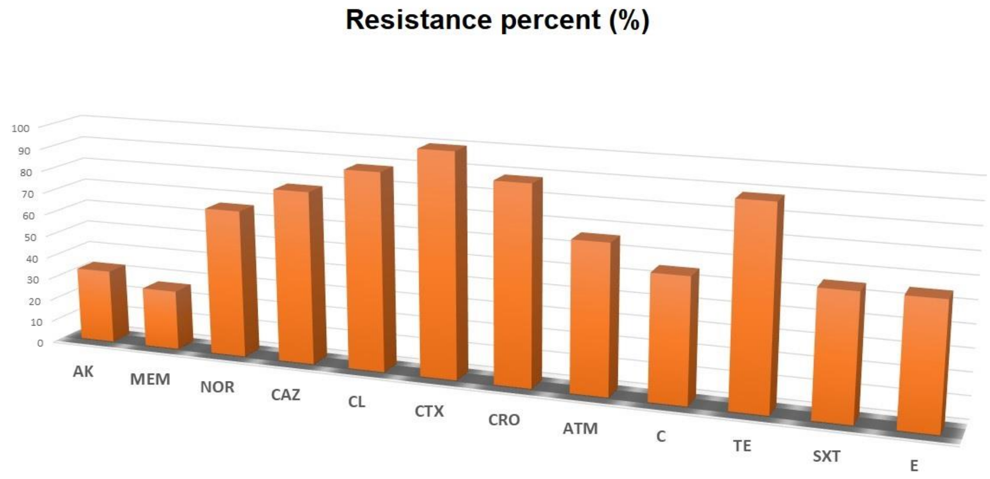

Figure S1. Antimicrobial resistance profile of *K. pneumoniae* isolated from animal sources; AK: Amikacin, NOR: Norfloxacin, TE: Tetracycline, CTX: Cefotaxime, CRO: Ceftriaxone, CAZ: Ceftazidime, C; Chloramphenicol, ATM: Aztreonam, SXT: Sulfamethoxazole-Trimethoprim, CL: Cephalexin, E: Erythromycin, and MEM: Meropenem.

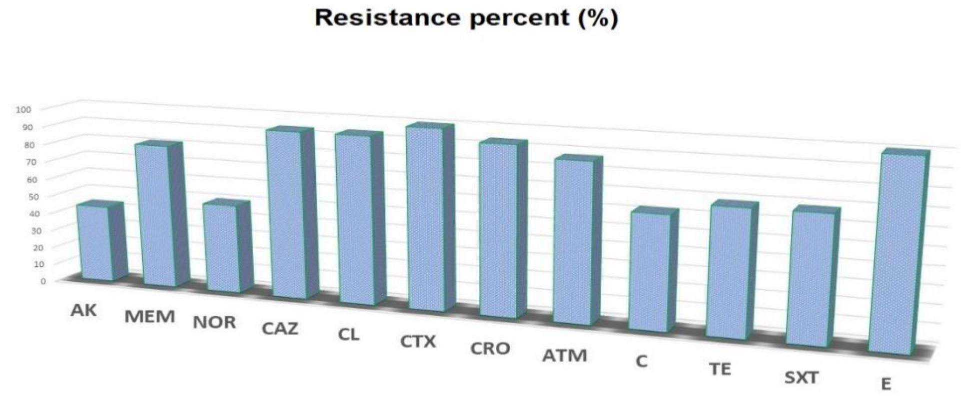

Figure S2. Antimicrobial resistance profile of *K. pneumoniae* isolated from human sources (clinical samples); AK: Amikacin, NOR: Norfloxacin, TE: Tetracycline, CTX: Cefotaxime, CRO: Ceftriaxone, CAZ: Ceftazidime, C; Chloramphenicol, ATM:

Aztreonam, SXT: Sulfamethoxazole-Trimethoprim, CL: Cephalexin, E: Erythromycin, and MEM: Meropenem.

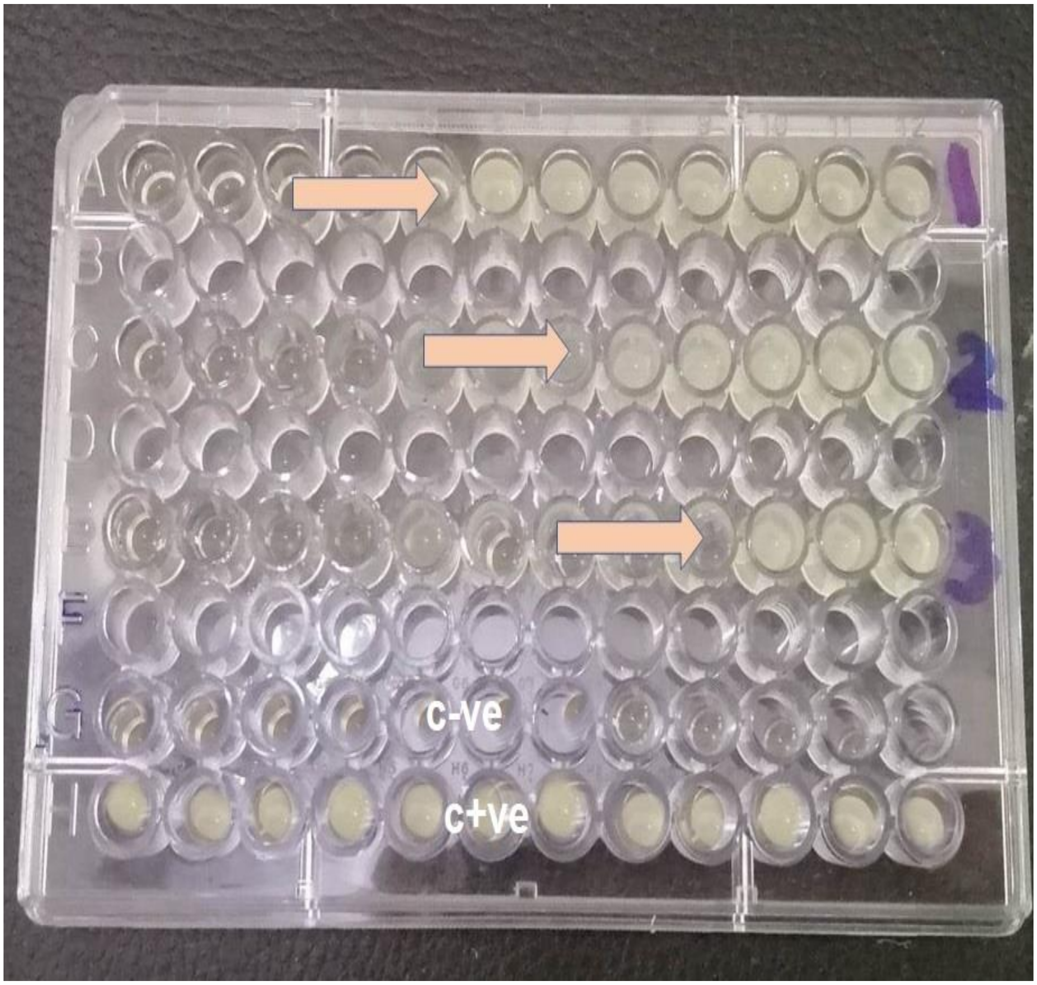

Figure S3. Minimum inhibitory concentrations (refers by arrow) of thyme (1), tea tree (2) and amikacin (3) against *K. pneumoniae* isolate using a 96-well microtiter plate. c+ve: control positive isolate without any treatment, C-ve: pure broth without any inoculum nor treatment.

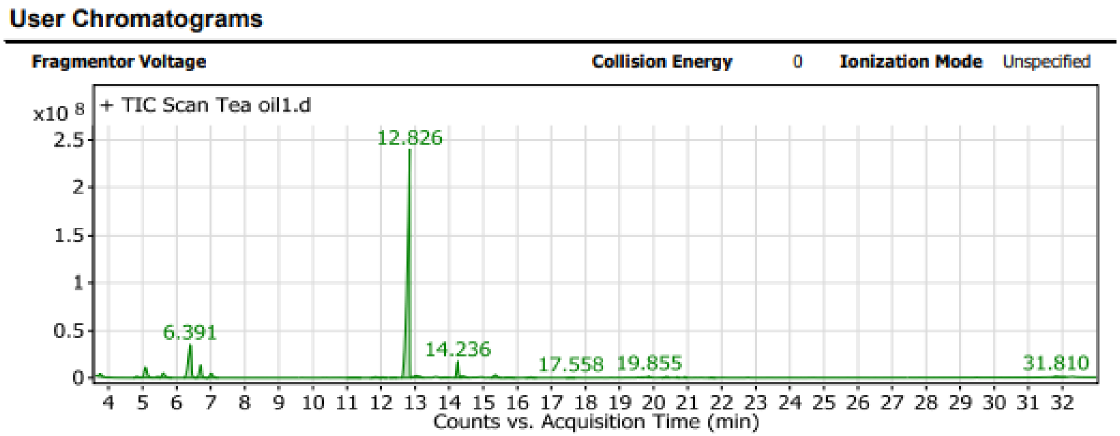

Figure S4. GC chromatography photo of tea tree oil extract

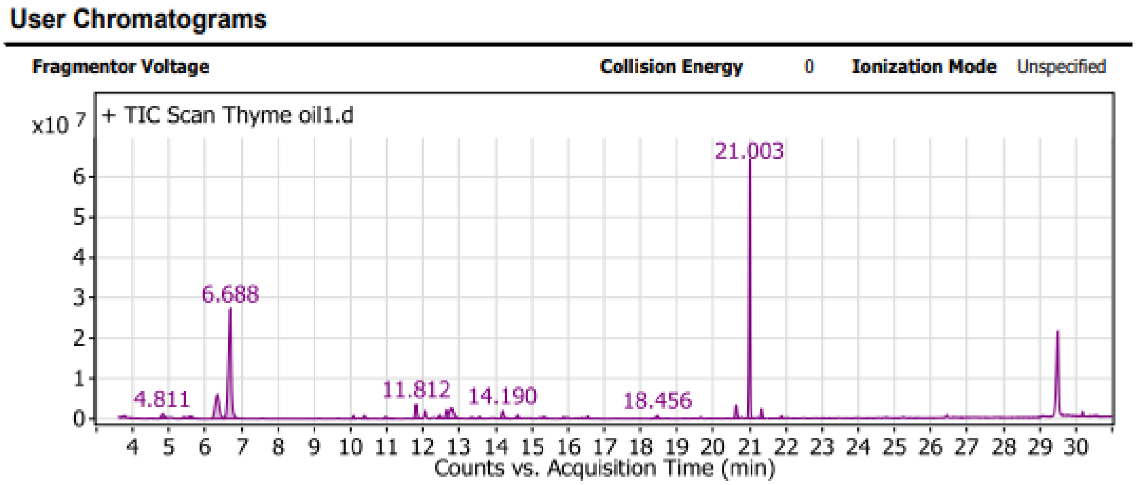

Figure S5. GC chromatography photo of thyme oil extract

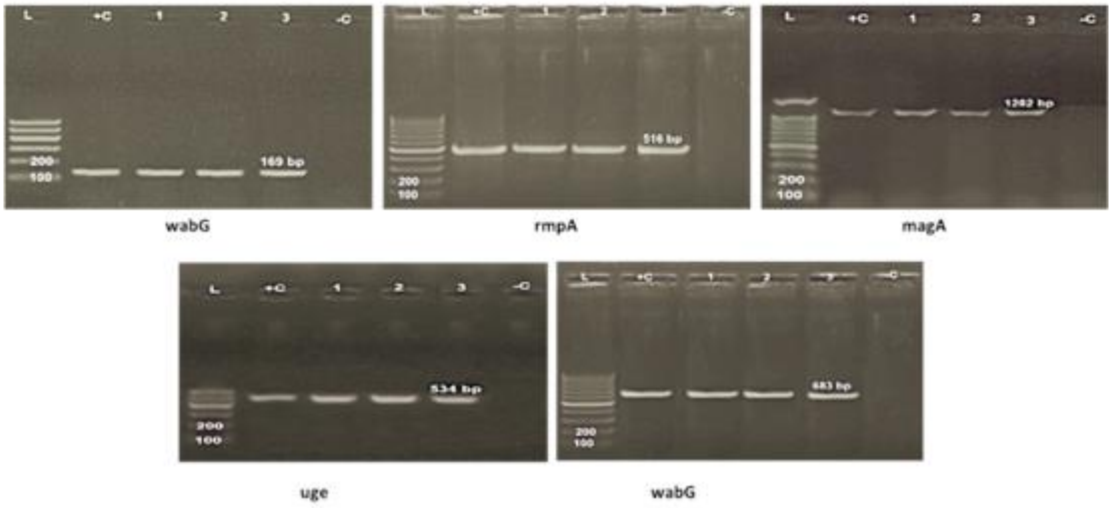

Figure S6. Agarose gel electrophoresis of the products amplified with polymerase chain reaction (PCR) using the specific primers for *Klebsiella pneumoniae* different gene; Lane +C: Positive control, Lane -C: Negative control, Lanes 1-3: tested isolates.

Table S1. ΔG kcal/mol of effective oils against the next target sites.

Tested compounds	Docking (Affinity) score (kcal/mol)		
	FosAKP	*K. pneumoniae* K1	OmpK36
(-) β-Pinene	-3.54	4.63	4.63
β-Myrcene	-3.54	-4.43	-4.72
β-Terpinolene	-3.48	-4.11	-4.63
Limonene	-3.83	-4.06	-4.35
Trans-sabinene hydrate	-3.47	-4.00	-4.22
ɣ-Terpinene	-3.23	-4.19	-4.19
p-Cymene	-3.61	-4.12	-4.72
Cyclohexene, 4-methyl-3-(1-methylethylidene)	-3.51	-3.96	-4.70
α-Copaene	-3.47	-3.93	-4.40

α-Gurjunene	-3.46	-3.85	-4.11
α-Humulene	-3.56	-4.45	-4.30
2,6-Dimethyl-3,5,7-octatriene-2-ol	-3.49	-5.98	-4.14
Terpinen-4-ol	-5.94	-5.81	-6.25
trans-Caryophyllene	-3.80	-3.91	-4.09
Naphthalene, 1,2,3,5,6,8a-hexahydro-4,7-dimethyl-1-(1-methylethyl)-, (1S-cis)-	-3.52	-4.50	-4.39
α-Terpineol	-3.34	-4.41	-4.20
Calarene	-3.66	-4.14	-4.06
Δ-Cadinene	-3.50	-4.31	-4.45
1H-Cyclopenta[1,3]cyclopropa [1,2]benzene, octahydro-7-methyl-3-methylene-4-(1-methylethyl)-, [3aS-(3a.alpha.,3b.beta.,4.b	-3.40	-4.25	-4.26
Cis-calamenene	-3.29	-4.17	-4.10
7-Oxabicyclo[4.1.0]heptane, 1-methyl-4-(2-methyloxiranyl)	-6.17	-6.08	-6.89
Farnesol	-5.63	-5.26	-5.04
ledol	-3.62	-4.05	-4.53
(+) spathulenol	-4.54	-4.88	-6.24
1,4-dihydroxy-p-menth-2-ene	-6.08	-5.44	-6.02
2-Pentenal, 2-ethyl	-3.90	-4.37	-4.95
(-) spathulenol	-4.63	-4.26	-5.93
trans-Z-.alpha.-Bisabolene epoxide	-3.26	-6.03	-6.93
Hydroquinone	-2.87	-3.87	-4.06

Table S2. **The utilized primers and their sequences of target genes for conventional PCR**

Genes	Primers (5'-3')	Product size	Annealing (T°C)	Reference
16S rRNA	F: ATT TGA AGA GGT TGC AAA CGA T<br>R: TTC ACT CTG AAG TTT TCT TGT GTT C	130	60°C	(Turton et al., 2010)
magA	F: GGTGCTCTTTACATCATTGC<br>R: GCAATGGCCATTTGCGTTAG	1282 bp	60°C	(Turton et al., 2010)
wabG	F: ACCATCGGCCATTTGATAGA<br>R: CGGACTGGCAGATCCATATC	683 bp	58°C	(Turton et al., 2010)
wcaG	F: GGTTGGKTCAGCAATCGTA<br>R: ACTATTCCGCCAACTTTTGC	169 bp	58°C	(Turton et al., 2010)
rmpA	F: ACTGGGCTACCTCTGCTTCA<br>R: CTTGCATGAGCCATCTTTCA	516 bp	60°C	(Turton et al., 2010)
uge	F: GATCATCCGGTCTCCCTGTA<br>R: TCTTCACGCCTTCCTTCACT	534 bp	53°C	(Nakayama et al., 2002)

Table S3. GC-MS analysis of different bioactive compounds of tea tree oil extract

Peak	RT	Compound name	Formula	Area%
1	12.826	Terpinene-4-ol	$C_{10}H_{18}O$	65.63
2	4.811	Beta-Myrcene	$C_{10}H_{16}$	0.47
3	5.423	Limonene	$C_{10}H_{16}$	0.35
4	19.567	Farnesol	$C_{15}H_{26}0$	0.08
5	6.688	P-Cymene	$C_{10}H_{14}$	3.67
6	3.739	Beta-pinene	$C_{10}H_{16}$	0.76
7	14.236	Alpha-Terpineol	$C_{10}H_{18}O$	3.32

RT: Retention time, Area %: Relative concentrations

Table S4. GC-MS analysis of different bioactive compounds of thyme oil extract

Peak	RT	Compound name	Formula	Area
1	21.003	Thymol	$C_{10}H_{14}0$	34.33
2	21.33	Carvacrol	$C_{10}H_{14}0$	1.36
3	11.812	Linalool	$C_{10}H_{18}O$	2.61
4	6.688	P-Cymene	$C_{10}H_{14}$	31.51
5	30.184	Hydroquinone	$C_6H_6O_2$	0.52
6	20.636	Eugenol	$C_{10}H_{12}O_2$	2.29
7	18.456	Caryophyllene oxide	$C_{15}H_{24}0$	0.97
8	16.416	Falcarinol	$C_{17}H_{24}O$	0.27
9	14.609	Carvone	$C_{10}H_{14}O$	0.62
10	13.554	Anethole	$C_{10}H_{12}O$	0.38

RT: Retention time, Area %: Relative concentrations

References

Nakayama, J., Kariyama, R., and Kumon, H. (2002). Description of a 23.9-kilobase chromosomal deletion containing a region encoding fsr genes which mainly determines the gelatinase-negative phenotype of clinical isolates of Enterococcus faecalis in urine. *Appl Environ Microbiol* 68, 3152–5. doi: 10.1128/AEM.68.6.3152-3155.2002

Turton, J. F., Perry, C., Elgohari, S., and Hampton, C. V (2010). PCR characterization and typing of Klebsiella pneumoniae using capsular type-specific, variable number tandem repeat and virulence gene targets. *J Med Microbiol* 59, 541–547.
